# Potent inhibition of nitroglycerin bioactivation by diphenyleneiodonium (DIP)

**DOI:** 10.1186/2050-6511-14-S1-P49

**Published:** 2013-08-29

**Authors:** Regina Neubauer, Andrea Neubauer, Gerald Wölkart, Christine Schwarzenegger, Barbara Lang, Kurt Schmidt, Michael Russwurm, Doris Koesling, Antonius CF Gorren, Astrid Schrammel, Bernd Mayer

**Affiliations:** 1 Department of Pharmacology and Toxicology, Karl-Franzens-Universität Graz, 8010 Graz, Austria; 2 Department of Pharmacology and Toxicology, Ruhr-Universität Bochum, 44789 Bochum, Germany

## Background

Aldehyde dehydrogenase-2 (ALDH2) catalyzes vascular bioactivation of the antianginal drug nitroglycerin (GTN) to yield nitric oxide (NO) or a related species that activates soluble guanylate cyclase (sGC), resulting in cGMP-mediated vasodilation [1]. Accordingly, established ALDH2 inhibitors attenuate GTN-induced vasorelaxation *in vitro* and *in vivo*. However, the ALDH2 hypothesis has not been reconciled with early studies demonstrating potent inhibition of the GTN response by diphenyleneiodonium (DPI) [2], a widely used inhibitor of flavoproteins, in particular NADPH oxidases. We addressed this issue and investigated the effects of DPI on GTN-induced relaxation of rat aortic rings and the function of purified ALDH2.

## Results

**Figure 1:**
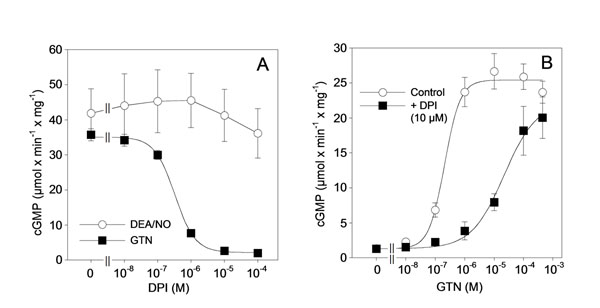
Effects of DPI on GTN-triggered activation of sGC in the presence of purified ALDH2. Purified sGC was incubated at 37°C for 10 min with 4 µg of ALDH2 in the presence of 0.5 mM [α-32P]GTP, 1,000 units/ml SOD, and 1 mM NAD^+^ with DPI, GTN or DEA/NO as indiacted, followed by isolation of ^32^P-cGMP and determination of radioactivity by liquid scintillation counting. A: Effects of the indicated concentrations of DPI on sGC activity determined in the presence of GTN or DEA/NO (1 µM each). B: Activation of sGC by increasing concentrations of GTN in the absnece and presence of 10 µM DPI. The data are mean values ± S.E.M. (n=3-4).

**Figure 2:**
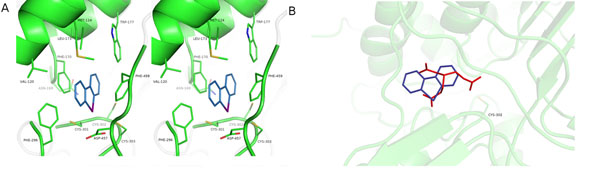
Model of DPI binding to ALDH2. A: Stereoscopic representation of the active site of ALDH2 (green) in complex with DPI (blue). The enzyme is shown as cartoon and active site residues and DPI are shown as sticks. B: Superposition of the modelled ALDH2-complex with DPI and the experimentally determined structure of GTN-bound ALDH2 (pdb-code 4fr8). The enzyme is shown as cartoon (green), Cys-302, DPI (blue), and GTN (red) are shown as sticks.

DPI (0.3 µM) inhibited the high affinity component of aortic relaxation to GTN without affecting the response to NO, indicating that the drug interfered with GTN bioactivation. As shown in Figure 1A, DPI inhibited bioactivation of GTN (1 µM) by ALDH2, assayed as activation of purified sGC, with an IC_50_ of 0.20±0.03 µM, whereas cGMP formation induced by the NO donor DEA/NO was not affected. DPI (10 µM) caused a pronounced right-ward shift of the GTN concentration response (Figure 1B), indicating that the drug acts in a GTN-competitive manner. The effect on GTN bioactivation was accompanied by GTN-competitive inhibition of 1,2-glycerol dinitrate formation (IC_50_ 0.21±0.04 µM in the presence of 2 µM GTN). DPI also inhibited the established dehydrogenase and esterase activities of ALDH2 with similar potency, again in a substrate-competitive manner. This was confirmed by molecular modeling, suggesting overlapping binding sites of DPI and GTN in the catalytic site of the enzyme (Figure 2). In contrast to ALDH2, ALDH1 and alcohol dehydrogenase were only partially (ALDH1) or not at all (ADH) inhibited by up to 0.1 mM DPI.

## Conclusion

The identification of DPI as potent ALDH2 inhibitor may have implications beyond the cardiovascular pharmacology of organic nitrates. A well established function of ALDH2 is detoxification of reactive aldehydes in liver and heart. NADPH oxidase activation has been implicated in both alcohol-induced liver injury and cardiac dysfunction. In view of the present results, showing that DPI potently inhibits the various enzymatic functions of ALDH2, it may be necessary to revise some of the earlier conclusions that are based on inhibitory effects of aryliodonium compounds.
